# Incidence and Risk Factors of Primary Arteriovenous Access Failure in Dialysis Patients: A National‐Based Retrospective Cohort Study

**DOI:** 10.1002/hsr2.70881

**Published:** 2025-09-21

**Authors:** Tarek A. Ghonimi, Abdullah Hamad, Musaab Elgaali, Mohamed Farid, Mohamed Ezat, Rania A/Aziz, Mohamed Amin, Hassan Al‐Malki, Mohamad Alkadi

**Affiliations:** ^1^ Division of Nephrology, Department of Medicine Hamad Medical Corporation Doha Qatar

**Keywords:** dialysis, End Stage Kidney Disease, failure, risk Factors, vascular access

## Abstract

**Background and Aim:**

The demand for hemodialysis (HD) among patients with end‐stage kidney disease (ESKD) is rising globally. A well‐functioning vascular access (VA) is crucial for effective dialysis therapy. This study aims to determine the incidence and risk factors of primary arterio‐venous access (AVA) maturation failure in HD patients.

**Methods:**

This retrospective cohort study included adult HD patients who underwent AVA creation between 01/01/2021 and 31/12/2023 in Qatar. Data, including demographics, medical comorbidities, AVA type, time to maturation, and incidence of AVA failure, were obtained from a national electronic health record system.

**Results:**

Among 242 AVA creations, the primary AVA failure rate was 28%, with an incidence of 9.3 per 100 cases per year. Failure was significantly higher in older patients (*p* < 0.001), those with diabetes mellitus (*p* = 0.03), atherosclerosis (*p* = 0.02), and lower systolic and diastolic blood pressures (*p* = 0.02 and *p* < 0.001, respectively). Statin use was higher in patients with matured AVA (68.9% vs. 45.5%; *p* < 0.001). Multivariate analysis identified diabetes mellitus [OR: 3.672 (95%CI: 1.532–8.801); *p* = 0.004], atherosclerosis [OR: 2.348 (95%CI: 1.001–5.504); *p* = 0.002], and age [OR: 1.036 (95%CI: 1.010–1.064); *p* = 0.007] as risk factors for failure. Statin use [OR: 0.167 (95%CI: 0.080–0.347); *p* < 0.001] and higher systolic blood pressure [OR: 0.989 (95%CI: 0.966–1.000); *p* = 0.05] were protective factors.

**Conclusion:**

Primary AVA failure in hemodialysis patients in Qatar is notably high, with advanced age, diabetes, atherosclerosis, and low blood pressure increasing risk. Statin use and higher systolic blood pressure provide protective effects. These findings highlight the need for managing risk factors to improve AVA maturation success and enhance dialysis outcomes.

## 1 Introduction

Chronic kidney disease (CKD) is a prevalent non‐communicable disease afflicting over 650 million people and resulting in more than 1.2 million deaths in 2017 worldwide [1]. The global prevalence of e (CKD) has been increasing, with significant variability across different regions and countries. The global median prevalence of CKD is 9.5%, with higher rates observed in Eastern and Central Europe (12.8%) [2]. This rise is partly attributed to the aging global population, which leads to a higher number of patients progressing to end‐stage kidney disease (ESKD) and an increased demand for hemodialysis (HD) therapy. In fact, by 2030, the worldwide use of kidney replacement therapy (KRT) is projected to exceed 5.4 million people [3].

Vascular access (VA) is the lifeline for HD patients, and includes central venous catheter (CVC) and arteriovenous access (AVA) such as arteriovenous fistula (AVF) and arteriovenous graft (AVG). Arteriovenous fistulas (AVFs) remain the preferred form of vascular access among hemodialysis patients, largely due to their extended longevity, reduced rates of infection and mechanical complications, and cost‐effectiveness in long‐term care [4, 5]. A large‐scale Japanese cohort also demonstrated a survival advantage in patients utilizing AVFs [6] In contrast, arteriovenous grafts (AVGs) are more susceptible to early thrombosis, often within the first year, and typically demand more frequent interventions to ensure continued function [7]. However, primary failure of AVF remains a major issue hindering access survival for HD patients [8]. Based on previous studies, several clinical factors have been identified as being associated with a higher early failure rate of AVF, such as old age, diabetes mellitus, cardiovascular disease, etc [9, 10, 11]. Of note, these risk factors are also common for mortality in HD patients [12]. Recent investigations have established an association between patency loss of AVA and mortality among hemodialysis patients [13, 14]. Multiple regional and systemic abnormalities in the setting of uremia might influence the development of eventual AVF failure. It has been suggested that various pathogenic conditions may predispose the vessel wall to inward remodeling and stenosis after AVF creation, including chronic inflammation, endothelial dysfunction, lipidemia, hyperparathyroidism, hyperphosphatemia, and hypercalcemia [15]. Thrombotic events represent the predominant mechanism underlying both early AVF failure and long‐term vascular access loss, accounting for a substantial proportion of permanent access complications. Thrombosis accounts for 65% to 85% of cases of permanent access loss [16]. Early thrombosis occurs in 5% to 30% of all created fistulae within 24 hours [17]. This study aims to determine the incidence of primary vascular access failure among hemodialysis patients in Qatar and to identify and analyze the associated risk factors.

## 2 Materials and Methods

This retrospective study targeted hemodialysis (HD) patients who underwent arteriovenous access (AVA) creation between January 1, 2021, and December 31, 2023, in dialysis centers across Qatar.

### 2.1 Objectives

The primary objectives of the study were:

- To determine the incidence of primary AVA failure among HD patients in Qatar.
- To analyze patient‐specific factors associated with primary AVA failure, including demographic, clinical, and laboratory characteristics.
- To compare patient characteristics between the two study groups: those with mature and functioning AVA and those with primary AVA failure.
- To explore the impact of clinical management and surgical factors on AVA outcomes.

### 2.2 Study Population

The study included all adult chronic HD patients from four dialysis centers in Qatar. These centers comprised the main and largest dialysis facility in Qatar, Fahd Bin Jassim Kidney Center, as well as three smaller units located in Wakra, Shahaniya, and Shamal.

### 2.3 Inclusion Criteria

Patients were included if they met the following criteria:

- Aged 18 years or older.
- Had been on HD for more than 3 months.
- Had undergone AVA creation (arteriovenous fistula [AVF] or arteriovenous graft [AVG]) during the study period.

### 2.4 Exclusion Criteria

Patients were excluded if they:

- Permanently relocated.
- Refused to use the AVA.
- Died or underwent transplantation before using the AVA created.

### 2.5 Consent Statement

The study was conducted in accordance with the Declaration of Helsinki and approved by the Institutional Review Board (IRB) at Hamad Medical Corporation (MRC‐01‐21‐994). Given the retrospective nature of the study, which involved reviewing data from an electronic database, informed consent from participants was not obtained. All data were anonymized to protect the confidentiality and privacy of participants, in strict adherence to ethical standards and guidelines.

### 2.6 Study Design

The included population was divided into two groups:

- Patients with matured and functioning AVA
- Patients with primary AVA failure.

A comparison of patient characteristics between these groups was performed. Finally, multivariable logistic regression models were utilized to identify patient factors independently associated with primary AVA dysfunction.

### 2.7 Arteriovenous Access Pathway

Eligible patients were evaluated by a vascular access coordinator. A referral was then arranged for a vascular surgeon to perform a detailed assessment, including clinical examination and Doppler ultrasound. Based on the assessment, a plan was developed regarding the type and location of AVA. Surgery was scheduled as a day‐care procedure for stable patients or after admission for patients requiring special care (e.g., those with cardiac conditions or on anticoagulants). Post‐surgery care was provided through the vascular clinic, in collaboration with dialysis vascular access coordinators.

### 2.8 Assessment and Management of AVA Maturation and Primary Failure

Primary AVA failure was defined as thrombosis or the inability to cannulate the AVA within 3 months of its creation [18]. Cases where the AVF did not appear to mature after 3 months, based on visual inspection, were referred to vascular surgery for further assessment. This included ultrasound evaluation of hemodynamic parameters, such as AVF blood flow, to determine if the AVF required additional time for maturation or failed to mature and was likely to thrombose or exhibit low flow volume.

### 2.9 Data Collection

All data were collected from the national electronic data system (Cerner) and included the following:

- **Demographic Data**: Age, gender, and ethnic background.
- **Clinical Data**: Body mass index (BMI), systolic and diastolic blood pressure, smoking status, dialysis vintage, primary causes of end‐stage kidney disease (ESKD), medications, and comorbidities such as diabetes, hypertension, dyslipidemia, atherosclerosis, coronary artery disease, and congestive heart failure. Additionally, data on the vascular surgeon performing the AVA creation and the time to AVA maturation were recorded.
- **Laboratory Data**: Serum potassium, albumin, hemoglobin, platelets, corrected calcium, phosphorus, parathyroid hormone (PTH), hemoglobin A1c (HbA1c), total cholesterol, and low‐density lipoprotein (LDL).

### 2.10 Definitions

- **Atherosclerosis**: Defined as a documented history of cardiovascular disease, including coronary artery disease, heart failure, stroke, or peripheral vascular disease.
- **Systolic and Diastolic Blood Pressure**: Assessed as continuous variables, based on the average of routine measurements obtained during dialysis sessions in the 6 months preceding AVA creation.

### 2.11 Statistical Analysis

Deidentified data were entered electronically and transferred to an Excel sheet for data analysis, which was conducted using SPSS version 21.0 (IBM Corp., Armonk, NY, USA). Descriptive statistics were used to summarize categorical and continuous variables. Frequency and proportion were calculated for categorical data, and mean ± standard deviation (SD) was used for continuous variables.

Comparisons between groups were made using an independent t‐test for continuous variables and a Chi‐square test for categorical variables. Univariate binary logistic regression analysis was performed to assess the association between each risk factor and the outcome of primary arterio‐venous access (AVA) failure. The multivariate binary logistic regression model, including confounders with significant effects (*p* < 0.05) from univariate analysis, was used to identify independent predictors of AVA failure.

Kaplan‐Meier survival analysis was used to assess the time to maturation of mature vascular access, with statistical significance evaluated using the log‐rank test.

A significance level of *p* ≤ 0.05 was considered statistically significant for all tests, and all tests were two‐sided. All analyses were performed using SPSS version 21.0.

Statistical Terms and Abbreviations

- *P*‐value: Probability of obtaining the observed results, or more extreme results, assuming the null hypothesis is true.
- Odds Ratio (OR): A measure of association between a risk factor and the likelihood of an outcome occurring.
- Confidence Interval (CI): A range of values within which the true parameter value is likely to lie, with a specified level of confidence.
- Kaplan‐Meier: A statistical method used for estimating the survival function from lifetime data.

### 2.12 Subgroup Analysis

Subgroup analyses were performed to assess potential differences in outcomes based on key variables such as age (≤ 60 vs. > 60 years), gender, comorbidities (e.g., diabetes, hypertension, coronary artery disease), smoking status, and primary kidney disease type. Additional exploratory analyses evaluated the effect of statin use, previous AVA creation, and access type on AVA failure rates. Statistically significant factors (*p* < 0.05) are presented in Table 1.

**Table 1:** Baseline characteristics of different patient groups at the time of entry.

	Total VA *N* = 242 (%)	Matured AVA *N* = 174 (%)	Primary AVA failure *N* = 68 (%)	*P*
Age	55.9 ± 14.8	54.0 ± 14.5	60.8 ± 14.8	0.001
Follow up/months (mean ± SD)	19.4 ± 10.1	19.4 ± 10.1	19.3 ± 10.2	0.97
≤ 60 years	146 (60.3)	116 (66.6)	30 (44.1)	0.001
> 60 years	96 (39.7)	58 (33.4)	38 (55.8)	
Gender				
Male	172 (71)	128(73.5)	44 (64.7)	0.17
Female	70 (28.9)	46 (26.4)	24 (35.2)	
Nationality				
Native	105 (43.4)	75 (43.1)	30 (44.1)	0.33
Arabic	69 (28.5)	43 (24.7)	26 (38.2)	
Non‐Arabic	68 (28.1)	56 (32.1)	12 (17.6)	
BMI	28.1 ± 7.5	28.2 ± 7	27.6 ± 8.7	0.53
Smoking	58 (24.8)	46 (26.4)	12 (17.6)	0.12
Co‐morbidities				
− Diabetes	168 (69.7)	113 (64.9)	55(80.8)	0.03
− HTN	224 (92.9)	159 (91.3)	65 (95.5)	0.32
− CAD	77 (32)	50 (28.7)	27 (39.7)	0.11
− Atherosclerosis	43 (17.7)	22 (12.6)	21 (30.8)	0.001
− CHF	41(17)	28 (16)	13 (19.1)	0.59
− Malignancy	17 (7.1)	12 (6.8)	5 (7.3)	0.91
Primary kidney disease				
− Diabetes	94 (39)	66 (37.9)	28 (41.1)	0.69
− Hypertension	6 (2.5)	3 (1.7)	3 (4.4)	
− OU	8 (3.3)	7 (4.0)	1 (1.4)	
− GN	23 (9.5)	17 (9.7)	6 (8.8)	
− APKD	5 (2.1)	3 (1.7)	2 (2.9)	
− Uncertain	100 (41.5)	73 (41.9)	27 (39.7)	
− MM	1 (0.4)	1 (0.5)	0	
− Vasculitis	2 (0.8)	2 (1.1)	0	
− lupus nephritis	1 (0.4)	1 (0.5)	0	
− primary oxalosis	1 (0.4)	0	1(1.4)	
Access type				
AVF	222 (91.7)	158 (90.8)	64 (94.1)	0.60
AVG	20 (8.3)	16 (9.1)	4 (5.8)	
AVF site:				0.45
Distal	109 (49.1%)	75 (47.4)	34 (53.1)	
Proximal	113 (50.9%)	83 (52.5)	30 (46.8)	
Previous AVA	79 (32.8)	55 (31.6)	24 (35.2)	0.60
Pre‐dialysis follow up	130 (53.9)	89 (51.1)	41 (60.2)	0.16
Aspirin use	116 (47.9)	83 (47.7)	33 (48.5)	0.07
Statin use	151 (58.8)	120 (68.9)	31 (45.5)	0.001
Surgeon				
− X	118 (49.4)	88 (50.5)	30 (44.1)	0.39
− XX	104 (43.5)	74 (42.5)	30 (44.1)	
− Other	17(7.1)	10(16.3)	7 (10.2)	

Abbreviations: APKD, Adult polycystic kidney disease; CAD, coronary artery disease; CHF,congestive heart failure; GN, Glomerulonephritis; HTN, Hypertension; MM, multiple myeloma; N, numbers; OU,obstructive uropathy; SD, standard deviation.

### 2.13 Ethical Issue

The study was conducted in accordance with the Declaration of Helsinki and approved by the Institutional Review Board (IRB) at Hamad Medical Corporation (MRC‐01‐21‐994). As a retrospective study relying on data from an electronic database, informed consent was not required. All data were anonymized to ensure participant confidentiality and privacy, in line with ethical guidelines.

## 3 Results

**Figure 1:**
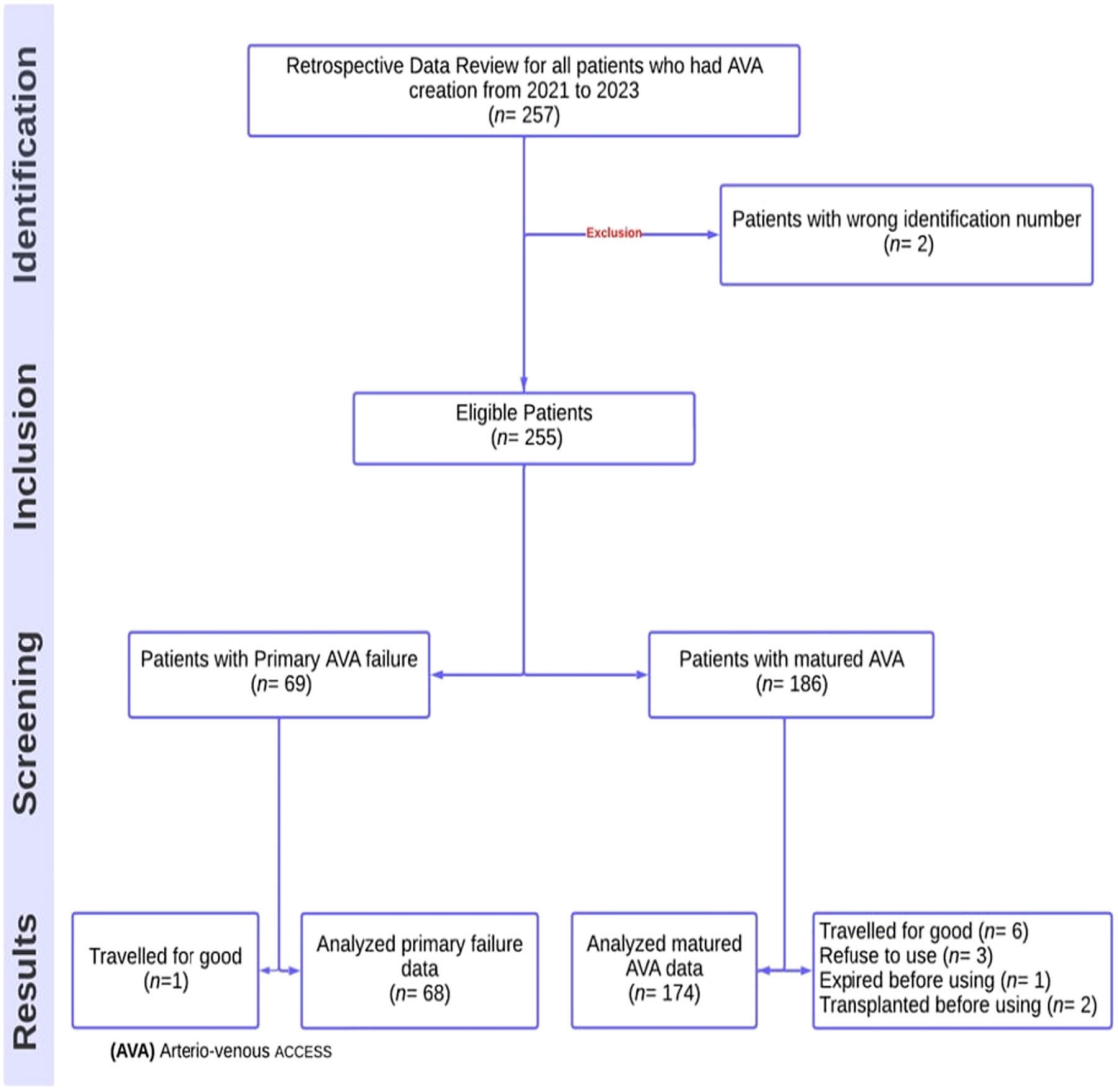
Flow Chart Diagram for all Patients with Arteriovenous access (AVA) creation between 2021 and 2023.

Between January 1, 2021, and December 31, 2023, a total of 257 patients underwent AVA creation. Two patients were excluded due to incorrect identification numbers, leaving 255 eligible for the study. Of these, 15 patients were excluded for various reasons, resulting in 242 patients meeting the inclusion criteria and being included in the final analysis. Among them, 68 patients experienced primary AVA failure, while 174 had mature AVAs, as illustrated in Figure 1.

### 3.1 Baseline Characteristics of the Study Groups

The prevalence of primary AVA failure was 28%, with an incidence of 9.3/100 cases/year. The median follow‐up period was 21 months, with a 25–75% quartile of 11–28 months. The mean age was 55.9 ± 14.8 years, with 146 (60.3%) ≤ 60 years old and 96 (39.7%) > 60 years old. One hundred seventy‐two patients were males (71%), while 70 (29%) were females.

Compared to patients with mature AVA, those with failed AVA were significantly older (60.8 ± 14.8 years vs. 54.0 ± 14.5 years; *p* = 0.001). Additionally, diabetes was significantly more prevalent in the AVA failure group than in the mature AVA group (80.8% vs. 64.9%; *p* = 0.03). Atherosclerosis was also more common in the AVA failure group compared to the mature group (30.8% vs. 12.6%; *p* = 0.001). Interestingly, patients on statins had a significantly higher rate of functioning mature AVA compared to those not using them (68.9% of patients vs. 45.5%, *p* = 0.001). There was no significant difference between the two groups regarding gender, ethnic groups, and BMI. More details are provided in Table 1.

### 3.2 Clinical and Laboratory Characteristics of the Studied Groups

Patients with primary vascular access failure had significantly lower systolic and diastolic blood pressure than those with mature access (systolic BP 132.5 ± 19 mmHg vs. 139.6 ± 20.5 mmHg and diastolic BP 72.1 ± 11.5 mmHg vs. 78.4 ± 12.1 mmHg, *p* = 0.02 and *P* = < 0.001, respectively). No significant differences were found between the two groups regarding HbA1c, corrected calcium, phosphorus, PTH, hemoglobin, platelets, serum albumin, LDL, and total cholesterol levels, as shown in Table 2.

**Table 2:** Clinical and laboratory characteristics of different patient groups.

parameter	All VA *N* = 242	Matured VA *N* = 174	Primary VA failure *N* = 68	*p*
Systolic blood pressure	137.620.5	139.6 ± 20.5	132.5 ± 19.2	0.02
Diastolic blood pressure	76.6 ± 12.3	78.4 ± 12.1	72.1 ± 11.5	< 0.001
Dialysis vintage/days	696.7 ± 1129.6.	723 ± 1194.5	630.1 ± 950.5	0.56
Hb A1c%	6.2 ± 1.6	6.1 ± 1.7	6.3 ± 1.2	0.42
Corrected Ca mmol/l	2.24 ± 0.16	2.2 ± 0.16	2.3 ± 0.16	0.43
Phosphorus mmol/l	1.6 ± 0.5	1.7 ± 0.5	11.6 ± 0.4	0.17
PTH pg/l	452.9 ± 444.7	451.2 ± 439.9	457.1 ± 460	0.93
HB gm/dl	10.9 ± 1.7	10.9 ± 1.9	11.1 ± 1.2	0.31
plateletsx10^3/uL	221.1 ± 71.7	224.7 ± 75	212 ± 62	0.22
Cholesterol mmol/l	3.4 ± 0.9	3.37 ± 0.91	3.53 ± 1.13	0.26
LDL mmol/l	1.6 ± 0.7	1.6 ± 0.6	1.7 ± 0.9	0.41
Albumin g/l	33.3 ± 4.7	33.1 ± 4.8	34.0 ± 3.6	0.18

Abbreviation: Ca, calcium; HB, hemoglobin, HbA1c, hemoglobin A1c; LDL, low density lipoprotein; N, Numbers; PTH parathyroid hormone.

### 3.3 Univariate and Multivariate Regression Analysis

The six variables—age, diabetes, statin use, atherosclerosis, systolic blood pressure, and diastolic blood pressure—were selected for the multivariate analysis based on their significant effects observed in the univariate analysis (*p* < 0.05). (see Table 3 for more details).

**Table 3:** Regression analysis of risk factors for AV access failure.

	Unadjusted Odds ratio	95% CI	*p*	Adjusted Odds ratio	95% CI	*p*
Age	1.034	1.013–1.055	0.002	1.036	1.010–1.064	0.007
Diabetes	2.134	1.079–4.224	0.03	3.672	1.532–8.801	0.004
Statin use	0.326	0.181–0.587	< 0.001	0.167	0.080–0.347	< 0.001
Atherosclerosis	2.172	1.063–4.438	0.03	2.348	1.001–5.504	0.002
Systolic blood pressure	0.983	0.969–0.997	0.02	0.989	0.966–1.000	0.05
Diastolic blood pressure	0.957	0.934–0.981	0.001	0.984	0.954–1.014	0.28

In the **univariate regression analysis**, older age (OR 1.034, 95% CI 1.013–1.055, *p* = 0.002), diabetes (OR 2.134, 95% CI 1.079–4.224, *p* = 0.03), statin use (OR 0.326, 95% CI 0.181–0.587, *p* < 0.001), and atherosclerosis (OR 2.172, 95% CI 1.063–4.438, *p* = 0.03) were significantly associated with AV access failure. Additionally, systolic blood pressure (OR 0.983, 95% CI 0.969–0.997, *p* = 0.02) and diastolic blood pressure (OR 0.957, 95% CI 0.934–0.981, *p* < 0.001) also showed significant associations.

In the **multivariate regression analysis**, after adjusting for confounders, older age (adjusted OR 1.036, 95% CI 1.010–1.064, *p* = 0.007), diabetes (adjusted OR 3.672, 95% CI 1.532–8.801, *p* = 0.004), and atherosclerosis (adjusted OR 2.348, 95% CI 1.001–5.504, *p* = 0.002) remained significant risk factors for AV access failure. Statin use (adjusted OR 0.167, 95% CI 0.080–0.347, *p* < 0.001) was significantly protective against failure. Systolic blood pressure (adjusted OR 0.989, 95% CI 0.966–1.000, *p* = 0.05) showed a marginally protective effect, while diastolic blood pressure did not remain significant (adjusted OR 0.984, 95% CI 0.954–1.014, *p* = 0.28).

### 3.4 Types of AV Access in Study Groups

The two types of AV access were arteriovenous fistula (AVF) in 222 cases (91.7%) and arteriovenous graft (AVG) in 20 cases (8.2%). All AVG placements were in proximal sites. Distal AVF was performed in 109 cases (49.1%), while proximal AVF was performed in 113 cases (50.9%). Although the percentage of primary AVF was higher in distal AVF than in proximal AVF (53.1% vs. 46.6%, respectively), there was no significant difference in fistula maturation between the two groups (*p* = 0.45). Refer to Tables 1 and 4 for more details.

**Table 4:** Types of V access in study groups.

Types of VA	All VA *N* = 242 (%)	Matured VA *N* = 174 (%)	Primary VA failure *N* = 68 (%)	*p*
Rt BC AVF	14 (5.8)	9 (5.1)	5 (7.3)	0.62
Rt RC AVF	12 (5)	9 (5.1)	3 (4.4)	
Lt BC AVF	94 (38.8)	72 (41.3)	22 (32.3)	
Lt RC AVF	60 (24.8)	38 (21.8)	22 (32.3)	
Lt snuff box AVF	15 (6.2)	12 (6.8)	3 (4.4)	
Lt ulno‐basilic AVF	4 (1.7)	3 (1.7)	1 (1.4)	
Rt Ulno Basilic AVF	2 (0.8	1 (0.5)	1 (1.4)	
Lt RC Mid forearm AVF	10 (4.1)	8 (4.5)	2 (2.9)	
Lt BC jump graft	10 (4.1)	7 (4.0)	3 (4.4)	
Rt BC jump graft	1 (0.4)	1 (0.5)	0	
Lt loop Brachio basilic loop graft	2 (0.8)	2 (1.1)	0	
Lt Redo	4 (1.7	2 (1.1)	2 (2.9)	
Lt Brachio‐subclvaian AVG	1 (0.4)	1 (0.5)	0	
Rt snuff box AVF	6 (2.5	4 (2.2)	2 (2.9)	
Lt Brachio‐axillary AVG	4 (1.7)	4 (2.2)	0	
Rt Brachio‐axillary AVG	1 (0.8)	0	1 (1.4)	
Rt brachiobasilic AVG	1 (0.8)	1(1.6)	0	
Left baslic vein transposition	1 (0.8)	1 (1.6)	1(1.4)	

Abbreviation: AVG, arteriovenous graft; Lt BC AVF, Left brachio‐cephalic arteriovenous fistula; Lt RC AVF, left radio‐cephalic arterio‐venous fistula; Rt BC AVF, right brachiocephalic arteriovenous fistula; Rt RC AVF right radio‐cephalic arteriovenous fistula.

### 3.5 Time to Vascular Access Maturation

**Figure 2:**
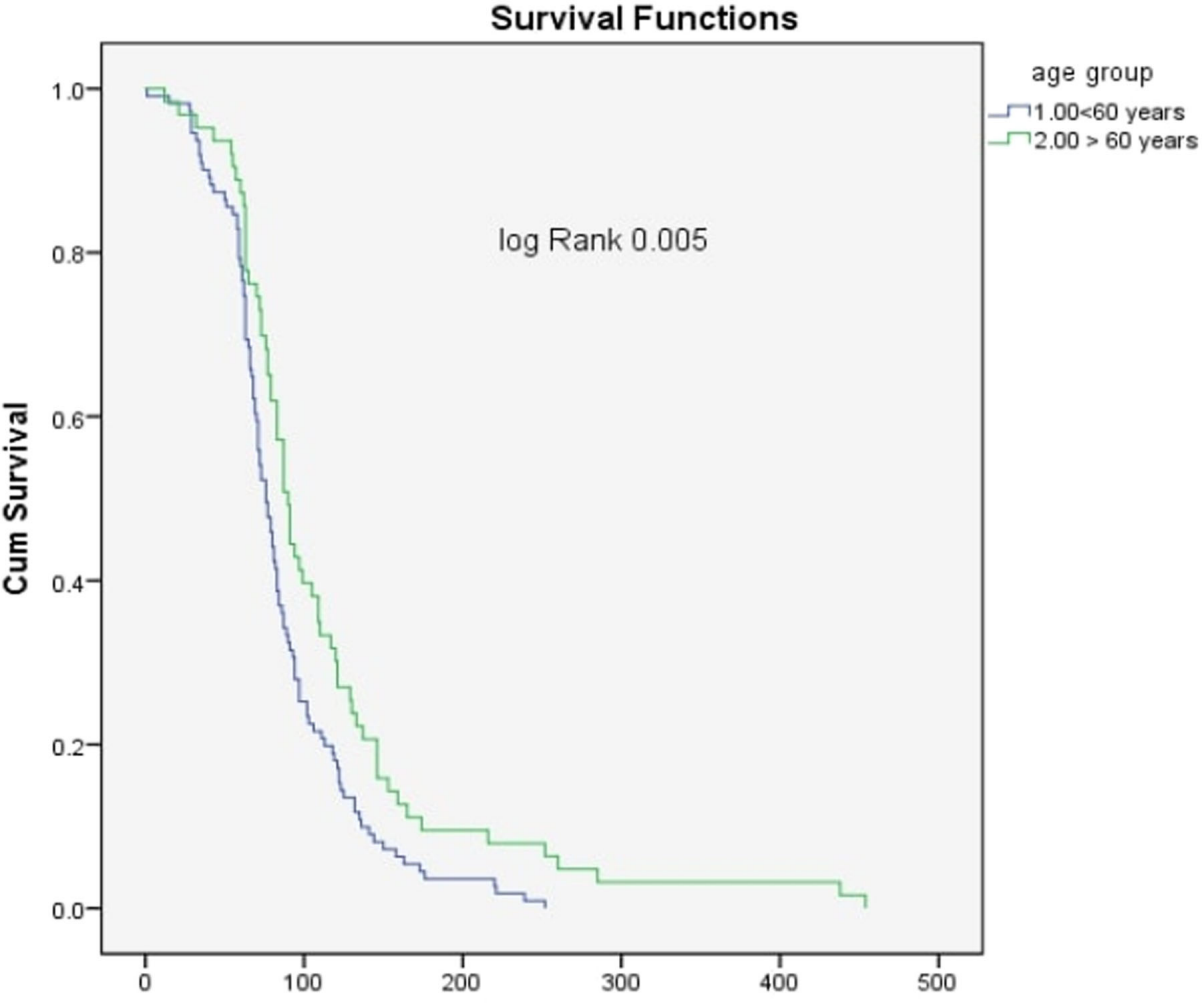
Time to AVF maturation/days.

In subgroup analysis, significantly longer days for AVA maturation were found in patients > 60 years of age, as seen in Figure 2 (log‐rank *p* = 0.005).

## 4 Discussion

Vascular access remains a significant economic, surgical, and logistical challenge for chronic kidney disease (CKD) patients and their healthcare providers. Among available vascular access options for dialysis, native AVFs are widely regarded as the most sustainable and economically viable choice [19, 20]. Since their introduction in the 1960s by Cimino and Brescia [21], techniques for AVF creation have progressed, contributing to improved clinical outcomes and broader surgical applications. This study aimed to identify the incidence and risk factors for primary vascular access failure in dialysis centers across Qatar.

In this study, we evaluated 242 patients who underwent AV access creation between January 2021 and December 2023 and found that the primary AV access failure rate was 28%, which is consistent with previous reports [22] (ranging from 27% to 37%). Primary AV access failure was significantly associated with older age, particularly in those over 60 years of age. Patients with primary AVA failure were significantly older (60.8 ± 14.8 years) compared to those with mature AVAs (54.0 ± 14.5 years), with a higher prevalence of diabetes and atherosclerosis. Diabetes and atherosclerosis were identified as significant risk factors for AVA failure, with odds ratios of 3.672 and 2.348, respectively. Interestingly, statin use was found to be a protective factor, with a significantly higher rate of functioning mature AVAs in statin users. Lower systolic and diastolic blood pressure were also linked to higher failure rates, with higher systolic blood pressure identified as a protective factor. These findings emphasize the importance of age, comorbidities, and blood pressure management in predicting vascular access outcomes.

In our study, primary AVA failure was significantly higher in older age, especially those > 60 years of age. However, this result contradicts a previous study, which concluded that age was not associated with an increased risk of primary failure [22], and another study by Lok et al. [23] showed equivalent survival fistula rates in elderly and young individuals. However, our study's result is consistent with a previous study [3], which concluded that the risk of primary arteriovenous fistula dysfunction was significantly higher in the elderly, and the odds ratio was 2.570, *p* = 0.02 in patients > 52 years. This result might reflect the high rate of atherosclerosis in these elderly patients, who could be at risk for maturation of vascular access.

Our study revealed a significant association between primary AVA failure and the presence of diabetes and atherosclerosis. Specifically, the multivariate analysis indicated that the odds ratio for primary AVA failure was 3.672 for diabetes and 2.348 for atherosclerosis. The literature presents conflicting evidence regarding the link between AV access failure and diabetes. While some researchers have identified an increased risk of primary AVF failure in diabetic patients [24, 25, 26], others have found no significant influence of diabetes on the primary failure rate [27, 28]. This discrepancy could be attributed to the elevated risk of vascular calcification and the prevalence of peripheral vascular disease in diabetic individuals, which predisposes them to primary access failure, particularly in distal AVFs [28, 29, 30].

A significant correlation was identified between lower systolic and diastolic blood pressures and increased primary AV access failure risk. Our findings suggest that preoperative blood pressure may influence AVA outcomes. Patients who experienced primary AVA failure demonstrated notably lower systolic and diastolic pressures before access creation., with *p*‐values of 0.02 and < 0.001, respectively. Multivariate analysis revealed that higher systolic blood pressure was a significant protective factor for primary AVA failure (OR 0.989). Supporting these findings, a previous study by Shajiee et al., [31] confirmed the impact of blood pressure on vascular access maturation, concluding that median systolic (SBP) and diastolic blood pressures (DBP) were significantly higher in functional vascular access compared to nonfunctional access (*p* < 0.05). Moreover, this observation supports earlier research, such as that by Feldman et al., who reported reduced maturation success among hypotensive patients [32]. Similarly, Thomsen et al. identified suboptimal outcomes in individuals with systolic pressures below 110 mmHg within a short follow‐up period, highlighting the importance of maintaining adequate perfusion pressure for vascular remodeling [33]. However, there are some opposing results compared to our findings. Siddiqui et al. reported no significant difference in blood pressure between functional and nonfunctional groups [34]. This suggests that other moderating and confounding factors may influence the relationship between blood pressure and arteriovenous fistula (AVF) maturation. Consequently, the effects of systolic blood pressure (SBP) and diastolic blood pressure (DBP) on AVF maturation should be evaluated in the context of these related factors. In fact, besides the role of blood pressure on arteriovenous fistula or graft maturation, the creation of dialysis access can lead to a decrease in blood pressure. Therefore, patients with lower preoperative blood pressure are more prone to postoperative hypotension [35]. Hypotension is reported to be one of the most important causes of AVF failure [36].

Some previous studies suggest that gender may influence AVF maturation. Siddiqui et al. found that males have a twofold higher maturation rate than females [34]. Similarly, Miller et al. discovered that AVF maturation is more successful in men than in women [37]. Lyem et al. also obtained similar results [38]. However, in the present study, we found no significant difference between genders regarding matured vascular access (VA) and primary access failure. This result is consistent with the findings of Fatemeh et al. [31], who also concluded that there was no significant difference between genders in functional and nonfunctional cases. A previous study found that primary AVF dysfunction was significantly associated with an increased platelet count and a low hemoglobin level [3]. Nevertheless, our study did not find any significant difference between functioning and nonfunctioning AV access in terms of hemoglobin A1c% (HbA1c%), corrected calcium (Corrected Ca), phosphorus, parathyroid hormone (PTH), hemoglobin (Hb), serum albumin, total cholesterol, and low‐density lipoprotein (LDL). However, our study revealed that using lipid‐lowering agents (statins) significantly decreased the risk of primary vascular access failure. There are conflicting data regarding the effect of cholesterol and lipid‐lowering therapy on the outcome of AV access

The potential role of lipid‐lowering therapy in AVA outcomes remains a subject of debate. However, a recent meta‐analysis found that elevated total cholesterol (TC) and low‐density lipoprotein cholesterol (LDL‐C) levels were significantly associated with increased risk of arteriovenous fistula (AVF) failure in patients undergoing hemodialysis, suggesting that lipid profile abnormalities may contribute to AVF dysfunction [39] However, other large observational studies have not confirmed a clear benefit of statin therapy in improving vascular access patency [40, 41]. Conversely, certain smaller trials have reported improved access longevity among statin users [42]. These mixed findings suggest that the impact of statins may depend on patient‐specific factors or concurrent therapies.

No significant effect of aspirin use as a risk factor for vascular access (VA) failure was identified in this study. Aspirin functions by irreversibly inhibiting platelet cyclooxygenase‐1 and ‐2 enzymes through acetylation, leading to decreased formation of prostaglandin precursors and the prostaglandin derivative thromboxane A2 [43]. Previous randomized controlled trials (RCTs) on the effectiveness of aspirin in preventing arteriovenous access failure have shown inconsistent results. Two small studies indicated the benefit of aspirin [44, 45], while two others found no significant treatment benefit for preventing arteriovenous access thrombosis and failure [46, 47]. However, data from the Dialysis Outcomes and Practice Patterns Study (DOPPS) demonstrated that consistent aspirin use had a beneficial effect on arteriovenous fistula (AVF) survival in incident hemodialysis patients [48].

In this analysis, we found no significant difference between matured and primary failed AVAs regarding the types and locations. However, there was a slight preference for proximal access over distal one. Previous results from the Dialysis Outcomes and Practice Patterns Study (DOPPS) [49] showed that successful AVF use was higher for upper‐arm AVFs than lower‐arm AVFs in the United States. Little difference was observed in Europe and Australia/New Zealand, while the opposite pattern was noted in Japan.

The role of vascular surgery was found to be nonsignificant in this study (*p* = 0.389). There are conflicting findings regarding the impact of the surgeon on the success of vascular access creation. Some authors argue that surgical skill is a crucial factor influencing the success of arteriovenous fistula (AVF) surgery [50, 51]. Previous studies have identified surgical experience as a statistically significant predictor of success in AV access surgery [52, 53]. Puskar et al. demonstrated that insufficient surgical experience contributed to AVF failure [54]. Additionally, Huijbregts et al. concluded that the probability of primary failure is strongly associated with the center of access creation, highlighting the significant role of the vascular surgeon's skill and decision‐making [55]. However, there are dissenting views. Data reported by Gundevia et al. and Weale et al. suggest that trainees can perform AVF procedures effectively with adequate supervision and appropriate case allocation. The fistula patency did not differ significantly between procedures performed by trainees and those performed by senior consulting surgeons in these two single‐center studies [56, 57]. Weale et al. propose that vascular access surgery can serve as a valuable training opportunity [57].

## 5 Strengths and Limitations

This study offers valuable insights into the incidence and risk factors for primary arteriovenous access (AVA) failure in a unique population of dialysis patients in Qatar. One of its strengths is the focus on this specific group, providing a detailed understanding of AVA outcomes in this setting. However, certain limitations should be considered. First, as a retrospective analysis utilizing data from our electronic records system, there is a possibility that some data may have been incomplete or missed. Second, the relatively small sample size, limited to hemodialysis patients from the main dialysis center and three smaller satellite units, may affect the generalizability of the findings and limit the number of AVA creation procedures included in the study. Third, the involvement of multiple investigators in data collection introduces a potential for bias. Finally, procedures performed during the same period for patients in the pre‐dialysis program were not included, which may also affect the comprehensiveness of the findings.

## 6 In Conclusion

The incidence of primary AV access failure is notably high. This study identified key risk factors contributing to this issue. The most significant predictors of primary vascular access failure were diabetes and atherosclerosis, while statin use was found to exert a protective effect. Early identification of these risk factors is crucial to mitigate the risk of AV access failure. This requires close collaboration between surgeons and nephrologists to enhance preoperative vascular screening and develop more precise criteria for evaluating the optimal approach for the primary creation of AV access. Additionally, extensive prospective trials are recommended to validate these findings.

## Author Contributions

**Tarek A. Ghonimi:** methodology, investigation, writing – original draft, formal analysis, data curation, supervision, writing – review and editing, conceptualization. **Abdullah Hamad:** methodology, validation, writing – review and editing. musaab elgaali: visualization, resources, data curation. **Mohamed Farid:** visualization, writing – review and editing. **Mohamed Ezat:** writing – review and editing, validation. **Rania A/Aziz:** data curation, resources. **Mohamed Amin:** writing – review and editing, data curation. **Hassan Al‐Malki:** supervision, writing – review and editing. **Mohamad Alkadi:** writing – review and editing, validation, formal analysis, supervision.

## Conflicts of Interest

The authors declare no conflicts of interest.

## Transparency Statement

The lead author Tarek A. Ghonimi affirms that this manuscript is an honest, accurate, and transparent account of the study being reported; that no important aspects of the study have been omitted; and that any discrepancies from the study as planned (and, if relevant, registered) have been explained.

## Author Statement

All authors have read and approved the final version of the manuscript. Dr. Tarek A. Ghonimi, the corresponding author, had full access to all of the data in this study and takes complete responsibility for the integrity of the data and the accuracy of the data analysis.

Note: An abstract based on this study was presented at the ASN Kidney Week on October 25, 2024 under a different author list, which included:

*Tarek A. Ghonimi, Abdullah I. Hamad, Musab Elgaali, Mohamed Y. M. Salih, Mohamed T. Abdellatif, Anees J. Alomari, Athar I. Hassan, Rania A. Ibrahim, and Hassan A. Al‐Malki*.

Some individuals who contributed to the abstract presentation were not involved in the final manuscript preparation, data analysis, or writing. These individuals have **no conflict of interest (COI)** related to this publication. All listed authors of the current manuscript meet the criteria for authorship and approve the final version.

## Data Availability

The datasets generated and/or analyzed for the current study are not publicly available due to privacy or ethical considerations, but they are available from the corresponding author upon reasonable request.
